# Nutritional and Technological Properties of Albino Peach Palm (*Bactris gasipaes*) from the Amazon: Influence of Cooking and Drying

**DOI:** 10.3390/foods12234344

**Published:** 2023-12-01

**Authors:** Stephanie Dias Soares, Orquídea Vasconcelos dos Santos, Leyvison Rafael Vieira da Conceição, Hilton Túlio Costi, José Otávio Carrera Silva Júnior, Francisco das Chagas Alves do Nascimento, Rosinelson da Silva Pena

**Affiliations:** 1 Graduate Program in Food Science and Technology, Federal University of Pará, Belém 66075-110, PA, Brazil; s203084@dac.unicamp.br (S.D.S.); orquideavs@ufpa.br (O.V.d.S.); 2 Faculty of Nutrition, Institute of Health Sciences, Federal University of Pará, Belém 66075-110, PA, Brazil; fcan@ufpa.br; 3 Faculty of Chemistry, Institute of Exact and Natural Sciences, Federal University of Pará, Belém 66075-110, PA, Brazil; rafaelvieira@ufpa.br; 4 Emílio Goeldi Museum, Coordination of Earth Sciences and Ecology, Belém 66077-830, PA, Brazil; tulio@museu-goeldi.br; 5 Faculty of Pharmacy, Institute of Health Sciences, Federal University of Pará, Belém 66075-110, PA, Brazil; carrera@ufpa.br; 6 Faculty of Food Engineering, Institute of Technology, Federal University of Pará, Belém 66075-110, PA, Brazil

**Keywords:** white peach palm, Amazon fruit, nutritional composition, technological properties, processing, Brazilian fruit, Arecaceae

## Abstract

This study aimed to subject the albino peach palm to cooking and drying processes and characterize the raw pulp (RP), cooked pulp (CP), raw pulp flour (RPF), and cooked pulp flour (CPF). The product’s chemical composition, bioactive compounds, and physicochemical, color, thermal, morphological, and functional–technological properties were evaluated. The proximate composition showed that carbohydrates were the main constituents of all the products (69.59–72.08 g/100 g). The cooking process decreased the lipids (10.21 to 8.63 g/100 g), dietary fiber (13.64 to 12.81 g/100 g), and total sugar content (59.18 to 49.10 g/100 g) of the CP. The colorimetric parameters indicated a significant browning of the CP and CPF, which can be attributed to the Maillard reaction and lipid oxidation. After cooking, the total phenolic compound and ascorbic acid content decreased in the pulp. The RPF and CPF displayed different thermogravimetric behaviors. The spectral patterns in the infrared region showed the characteristic bands of organic compounds that are present in the structure of starches. The scanning electron microscopy showed amyloplast and fiber bundles with starches in the RP and gelatinized starch granules in the CP and CPF. The RPF presented small and heterogeneous starch granules with isolated amyloplast. The RPF and CPF showed different granulometric patterns and technological indices. The results suggest that the pulp and flour from the pulp of albino peach palms can be exploited by the food, pharmaceutical, and biotechnological industries.

## 1. Introduction

The Amazon region is rich in native fruits of various species, which are of great importance for the local population’s culture, food, economy, and health [1]. These fruits have been a source of research related to micronutrients, bioactive compounds, antioxidant capacity, and biological activities [2,3,4]. More than 40% of the native Brazilian fruits are found in the Amazonian region, especially the northern region, which produces approximately 220 species of plants that produce fruit for human consumption. However, the Arecaceae family stands out with its fruits produced by palm trees, such as peach palm (*Bactris gasipaes*), *açaí* (*Euterpe oleracea*), *bacaba* (*Oenocarpus bacaba*), and *buriti* (*Mauritia flexuosa*). These species can be used by directly consuming its fruits and obtaining products and byproducts, such as drinks, creams, flour, and oils [5,6].

The peach palm fruit is known worldwide as “pupunha” (in Brazil), “pejibaye” (in Costa Rica), and “chontaduro” (in Colombia) [2,7,8]. This fruit has ovoid and globose anatomic characteristics, but it is best known for its different colorations of green (immature fruit), yellow, orange, and red, provided by the presence of carotenoid pigments. Recently, our research group presented research on an albino peach palm variety from the Amazon not previously explored or reported [3], corroborating what was reported by Cornelius et al. [9], who found the presence of peach palm fruits with yellow peel and white pulp for the first time.

The peach palm fruit needs to be cooked in water before consumption to inhibit anti-nutritional factors (trypsin inhibitors, phytates, and tannins), eliminate oxalate crystals present in the peel [10,11], and inactivate the pulp peroxidase enzyme (105 °C for 20 min), thus avoiding the irritation of the throat mucosa [12]. On the other hand, this fruit is considered a fruit with high nutritional value because it has a significant content of oil and proteins, and a high content of carbohydrates and insoluble dietary fiber [11,13,14]. In addition, the peach palm fruit has all the essential amino acids [15]. The flour and starch obtained from the pulp of peach palms (with or without peel) have been extensively studied for their nutritional, functional, and biotechnological properties. Reports have shown the biological potential of using these products to improve metabolic properties [16,17], to increase the bioaccessibility of carotenoids in foods [18,19], and to obtain a biodegradable film from starch [20].

The cooking process required for fruit consumption and the drying to obtain flour could lead to significant changes. These processes promote changes in food’s physical and chemical characteristics, such as browning and protein denaturation, nutritional composition, and cellular structure. In addition, these processes affect food products’ sensory and color characteristics [21,22]. Despite that, drying is an important process for food preservation, which ensures the product’s microbiological and chemical stability, promoting a longer shelf life [23,24,25].

Therefore, this work aimed to evaluate the chemical composition, bioactive compounds, and physicochemical, color, thermal, morphological, and functional–technological properties of albino peach palms before and after the cooking and drying processes. To the best of our knowledge, this work comprises the first report on the influence of the cooking and drying of albino peach palms.

## 2. Materials and Methods

### 2.1. Fruits

Five bunches (~20 kg each) of albino peach palm (~100 kg of fruits) were acquired at a fair located in the metropolitan region of Belém, the state of Pará, Brazil (latitude: −1.45502, longitude: −48.5024, 1°27′18″ S, 48°30′9″ W), and which were harvested during the period September to December 2020. This research was registered with access activity in the National System for the Management of Genetic Heritage and Associated Traditional Knowledge (SisGen, AA5BEE7). The voucher specimen was identified and deposited in the Herbarium “HF Professora Normélia Vasconcelos” at the Federal University of Pará (Belém, Brazil) and notified under register 4070.

### 2.2. Sample Preparation

At this stage, half of the fruits (with peel) were cooked in water in a pressure cooker for 15 min, according to the traditional procedure performed in Northern Brazil. The other half of the fruits were separated without cooking. Both fruit samples (raw and cooked) were peeled, and the pulp was separated from the seed using a stainless steel knife. The edible portion of peach palm (raw and cooked pulps) was crushed separately in a multiprocessor (Philco, 800 W, Manaus, Brazil) and then placed in plastic bags and packed in a vacuum sealer (Cetro, model DZ-280, São Paulo, Brazil). The samples were stored at −20 °C and wrapped in aluminum foil for protection against light until the analysis. To obtain raw and cooked peach palm flour, a part of the samples of their respective pulps was subjected to drying at 55 °C [14] in an oven with forced air circulation (Thoth, model 510.150, São Paulo, Brazil) until a constant weight was obtained (~26 h). The dried samples were ground in a Willey-type knife mill (Fortinox, model Start FT 50, São Paulo, Brazil) coupled to a ten-mesh sieve.

### 2.3. Physicochemical Characterization

The analyses of moisture, ash, total proteins (total nitrogen by the Kjeldahl method and conversion factor of 5.75), lipids, total titratable acidity (TTA; %citric acid), hydrogen potential, and total and reducing sugars were performed according to the Association of Official Analytical Chemists [26]. Dietary fibers (total and insoluble fractions) were determined using the enzymatic/gravimetric method [27]. The soluble dietary fiber content was determined by difference. Total carbohydrates were obtained by difference, and the caloric value was measured using the Atwater coefficients [28]. The water activity (a_w_) was determined with a direct measure on a water activity analyzer (Aqualab 4TEV, Decagon Devices Inc., Pullman, WA, USA).

The instrumental color parameters were analyzed in a digital colorimeter (Chroma Meter CR-300, Konica Minolta, Tokyo, Japan), using the CIELAB system to assess the chromaticity coordinates (L* for luminosity, a* for red color intensity, b* for yellow color intensity). The chromaticity (C*) and the Hue angle (*h°*) were calculated according to McLellan et al. [29]. To know how close a sample is to an ideal white, the whiteness index (WI) was calculated [30].

### 2.4. Mineral Composition

This analysis was performed according to Alves et al. [31]. A mass of 0.25 g was weighed for each sample in triplicate (*n* = 3) and digested with 5.0 mL of HNO_3_ 25% (*v*/*v*) and 5.0 mL of H_2_O_2_ 30% (*w*/*w*) in a microwave oven. The heating program consisted of the following three steps: 800 W, 180 °C for 10 min; 800 W, 180 °C for 15 min; and ventilation for 50 min. After digestion, the digested part and the residue (silicate compounds that were not digested) were quantitatively transferred to a 14 mL volumetric flask, and 10 mL of ultrapure water was added. The same procedure was used to prepare the analytical blank, without adding the sample. For the analysis of Al, As, Ba, Ca, Cd, Cr, Cu, Fe, Mg, Mn, P, Se, and Zn, an inductively coupled plasma optical emission spectrometer (iCAP 6500, Thermo Scientific, Cambridge, UK) was used. All solutions and dilutions were prepared with ultrapure water (resistivity 18.2 MΩ cm) obtained from a water purification system (Synergy UV, Millipore, Darmstadt, Germany). Nitric acid (Sigma-Aldrich, Darmstadt, Germany) was used at 65% *v*/*v*, previously purified by a distillation system (BSB 939-IR, Berghof, Eningen, Germany).

### 2.5. Ascorbic Acid, Phenolic Compound, Flavonoid, and Carotenoid Content

The titrimetric method quantified the ascorbic acid content with the reagent 2,6-dichlorophenolindophenol (DCFI) [26]. The total phenolic compound (TPC) content was spectrophotometrically quantified using the Folin–Ciocalteau reagent according to the colorimetry method described by Singleton and Rossi [32]. An analytical curve of gallic acid (1 to 100 mg/L) was used. The total flavonoid content was determined using the aluminum flavonoid complexation reaction method described by Pękal and Pyrzynska [33]. An analytical curve of quercetin in the 1.56 to 25 µg/mL range was used. Finally, the total carotenoid content was quantified with spectrophotometry using the method proposed by Godoy and Rodriguez-Amaya [34]; the sample was subjected to a measurement in the UV-Vis spectrophotometer at 450 nm. The total carotenoid content was calculated using the specific absorption coefficient of β-carotene in petroleum ether ($A_{1\mathrm{cm}}^{1\%}=2592$).

### 2.6. Granulometric Analysis

A digital electromagnetic stirrer (Bertel, model VP-01, São Paulo, Brazil) was used, with a constant stirring speed and frequency of 50/60 HZ. Each flour was sieved in a set of 9-, 20-, 28-, 60-, 80-, and 100-mesh sieves (with mesh openings of 2000, 850, 600, 250, 180, and 150 µm, respectively), and a pan, for 10 min [35]. The samples’ mass retained on the sieve and the initial mass were considered for calculation.

### 2.7. Thermogravimetric and Absorption Spectroscopy Analysis

Thermogravimetric analyses of the raw and cooked pulps and flour were performed with a thermobalance (Shimadzu, model DTG-60 H, Kyoto, Japan) under the following conditions: airflow of 60 mL/min, a heating ramp of 10 °C/min, in the temperature range of 20 to 800 °C, in an alumina crucible with a mass of 5 mg (±0.5).

In the infrared region, the absorption spectroscopy was carried out using the same samples with a spectrometer (Shimadzu Corporation IR Prestige 21 Cat. No. 206-73600-36, Kyoto, Japan) with records in the spectral frequency range of absorption from 4000 to 500 cm^−1^. The sample was incorporated into potassium bromide pellets, with Scan 100 and a resolution of 4 cm^−1^. All bands were analyzed by the software Origin 8.0.

### 2.8. Scanning Electron Microscopy

The micrographs were obtained using an electron microscope (TESCAN, model Mira3, Brno, Czech Republic) with an electron gun-type FEG (field emission gun). The samples were metalized with Au for 2.5 min for the deposition of a film on the sample, with a 10 to 15 nm thickness. The images were generated by detecting secondary electrons, using a voltage acceleration between 5 and 10 kV and working distances between 10 and 15 mm.

### 2.9. Functional Technological Properties

The functional technological properties: water solubility index (WSI), water absorption index (WAI), and oil absorption capacity (OAC), were performed and calculated according to Anderson et al. [36], with some modifications. The foam forming ability (FFA) and foam stability (FS) were determined according to Coffmann and Garcia [37].

### 2.10. Statistical Analysis

All analyses were performed in triplicate, and the results were expressed as mean ± standard deviation. Data were submitted for analysis of variance (ANOVA) and Tukey’s complementary test for means comparison (*p* ≤ 0.05) using the software the version number for software Statistica 10.0 (TIBCO Software Inc., Palo Alto, CA, USA).

## 3. Results and Discussion

### 3.1. Physicochemical Properties of Pulp and Flour

The proximate composition and physicochemical properties of the pulp and flour of the raw and cooked albino peach palm are presented in Table 1. The cooked pulp (CP) presented a higher moisture content than the raw pulp (RP), which could be attributed to the incorporation of water during cooking. On the other hand, the cooked pulp flour (CPF) reached a lower moisture content than the raw pulp flour (RPF), indicating that cooking favored the elimination of the water from the pulp during drying. The pulp moistures were similar to the values observed by Pires et al. [14] for fruits of the microcarp (62.43 g/100 g) and mesocarp (63.96 g/100 g) varieties. In turn, the moisture contents of the RPF and CPF were lower than the values found by the same authors for the pulp flour of the microcarp, mesocarp, and macrocarp varieties (9.60–12.58 g/100 g) obtained by convective drying at 55 °C. The differences observed may be attributed to the different peach palm varieties evaluated, the drying system used, and the origin of the fruits.

For the ash content, a significant difference was observed (*p* ≤ 0.05) between the pulps, indicating that cooking caused significant changes in this component. The ash contents found in both flour (2.12–2.21 g/100 g dm) allowed for the classification of these samples as having a low ash content (2–6 g/100 g) [38]. The results found for the RPF and CPF were close to those reported by Martínez-Girón et al. [39] for peach palm peel flour (1.95 g/100 g dm). On the other hand, the ash contents observed in the pulps (9.68–13.93 g/100 g dm) were higher than the values reported by dos Santos [3] for cooked albino peach palm (5.43 g/100 g dm) from other varieties.

Regarding proteins, the content observed in both flour was higher than in the respective pulps (*p* ≤ 0.05). This behavior can be attributed to the decrease in other flour constituents, such as total sugars. The protein content found in both albino peach palm flour (RPF and CPF) was higher than that found (8.64 g/100 g dm) for a peach palm by-product (stem portion) [40]. From a nutritional point of view, 100 g of the CP provided approximately 4% of the recommended daily protein intake for a healthy adult (body weight = 70 kg) and 8% for an elderly person. The CPF (100 g), in turn, contributed approximately 32 and 64% of the recommended daily protein intake for these respective populations [41]. Thus, peach palm flour can provide excellent protein levels, whether consumed directly or in culinary preparations fortified with this flour [18,42,43].

The lipid contents were statistically equal for the four samples (RP, CP, RPF, and CPF) (*p* > 0.05), which suggests that neither cooking nor drying caused the loss of this constituent. However, a tendency towards a reduction in the lipid content was observed in the CP and CPF, compared to the values of the RP and RPF, respectively, which can be attributed to a small loss of lipids due to leaching during cooking. The lipid values in the RP and CP (10.21 and 8.63 g/100 g dm) were within the range observed by Melo et al. [11] (4.21–21.74 g/100 g dm), but were higher than the values observed by Basto et al. [42] (6.6–7.5 g/100 g dm). In our recent study [3], we reported for the first time the fatty acid profile in peach palm oil, which is mainly composed of saturated fatty acids (51.75%), especially palmitic acid (49.71%)—with 42.29% being monounsaturated fatty acids and 4.86% being polyunsaturated fatty acids.

The carbohydrates were the major macronutrient in the pulps (RP and CP) and flour (RPF and CPF), and the values were not statistically different (*p* > 0.05), indicating that neither cooking nor drying caused the loss of this macronutrient. In turn, the total dietary fiber content was not statistically different (*p* > 0.05) between pulps, but the values were statistically different (*p* ≤ 0.05) between flours. For the total and insoluble fibers, a decreasing trend in the CP and CPF values was observed concerning the RP and RPF, which can be attributed to the leaching of these constituents. The insoluble dietary fiber represented 68.2%, 57.1%, 87.1%, and 73.6% of the total dietary fiber in the RP, CP, RPF, and CPF, respectively. These results corroborated those Pires et al. [14] reported for peach palm flour of the mesocarp variety. In our recent study [3] we found 10.4, 7.2, and 3.2 g/100 g dm of total, insoluble, and soluble dietary fiber, respectively, in the CP. Dietary fibers can act as functional foods that, when consumed regularly and combined with healthy lifestyle habits, are related to reducing insulin levels, managing cardiometabolic diseases, reducing cholesterol levels, and improving intestinal health [44,45,46]. Therefore, fiber-rich flour can provide functionality to food products [47].

A lower content of total sugars was observed in the CP and CPF compared with the respective RP and RPF (*p* ≤ 0.05). Similar behavior was observed for reducing and non-reducing sugar fractions. These results indicated that during cooking, the dissolution and leaching of sugars occurred, as the same was found by Giombelli et al. [40] for the by-product of heart of palm production (stem portion). The content of non-reducing sugars in the RP and CP was higher than that of reducing sugars. On the other hand, the content of reducing sugars in both flour was higher than that of non-reducing sugars, which suggests that, during the drying of the pulp at 55 °C, the depolymerization of non-reducing sugars may have occurred through the selective hydrolysis of glycosidic bonds [48].

A higher total energetic value (TEV) was observed for the RP (160.58 kcal/100 g wb) than for the CP (115.19 kcal/100 g wb) (*p* ≤ 0.05), while, for the RPF and CPF, the TEV was statistically equal (average TEV of 403.4 kcal/100 g wb) (*p* > 0.05). The TEV observed in both flour was higher than the values reported for wheat (366.6 kcal/100 g) and oat (363.7 kcal/100 g) flour [49], which indicates that the albino peach palm flour is an excellent source of calories, which can contribute to energy balance [50].

The data showed that the reduction in the moisture promoted by drying ensured the obtainment of flour with low a_w_ values (a_w_ < 0.25), capable of guaranteeing the microbiological stability of the products (a_w_ < 0.6). On the other hand, the a_w_ values in the RP and CP (0.98) were highly favorable to the activity of microorganisms, chemical and enzymatic reactions, and physical changes in the products [23,24,25]. The pH values indicated that the pulps and flour are products with low acidity (pH > 4.5). On the other hand, the total titratable acidity (TTA) of the RP (8.96 g citric acid/100 g wb) was approximately twice the observed value in the CP (4.48 g citric acid/100 g wb). This reduction can be attributed to the leaching of the acidic constituents during cooking [51], such as the tannins and phytates in peach palm fruits [10].

### 3.2. Color Parameters

The instrumental color results observed for the pulps and flour of the albino peach palm are shown in Table 2. The RP and RPF showed a different colorimetric pattern than the respective CP and CPF (*p* ≤ 0.05). On the other hand, the RP and RPF, as well as the CP and CPF, in general, showed the same colorimetric pattern (*p* > 0.05). Cooking caused a decrease in the brightness (L*) and whiteness index (WI) and an increase in the parameters b* (yellow color) and C* (chromaticity) of the pulp. The L* and WI values decreased significantly during cooking (*p* ≤ 0.05), but, during drying, no reduction in these parameters was observed (*p* > 0.05). Therefore, there occurred a browning of the pulp during cooking (Figure S1) which can be attributed to lipid oxidation and the Maillard reaction caused by the interaction between the amino acids and reducing sugars (glucose and fructose) of the pulp [40].

The increase in the b* value corroborated the difference observed in the color of the RP and CP (Supplementary Material Figure S1) and visually indicated that cooking the fruit promoted an increase in the concentration of the yellow pigments in the pulp. Despite the whitish visual aspect of the RP (Supplementary Material Figure S1), the b* values indicated the presence of yellow pigments, both in the RP (b* = +18.56) and in the RPF (b* = +16.0), which was confirmed by the values of the C* and *h°* that were attributed to the light yellow color of the RP (C* = 18.57 and *h°* = 91.35°) and RPF (C* = 15.17 and *h°* = 94.92°) [28]. In turn, the increase in the C* values indicated an increase in the intensity of the yellow color of the CP (C* = 25.93) and CPF (C* = 24.93).

### 3.3. Mineral Composition

Table 3 shows that Mg, P, and Ca were the major minerals in the pulps and flours of the albino peach palm, while Fe, Cr, Cu, and Mn were the main trace elements. Our research group [3] reported, for the first time, an elemental composition with Ca (1500 mg), P (860 mg), Mg (815.8 mg), Se (76.2 mg), Zn (45.3 mg), Fe (23.0 mg), Cu (13.9 mg), and Mn (7.2 mg) per kilogram of cooked albino peach palm (in dry matter). These values were generally lower than those observed in the CP, except for Cu, Se, and Zn. Variations in the mineral composition can be explained by differences in the soil composition, agricultural practices, and environmental conditions [52]. The contents of Cu, Fe, and Zn in the RP were higher than in fruits such as *jambo* (*Syzygium jambos*) (5.71 mg/kg for Cu), mangosteen (*Garcinia mangostana*) (5.71 mg/kg for Cu and 23.3 mg/kg for Fe), *muruci* (*Byrsonima scrispa*) (1.8 mg/kg for Cu and <0.2 mg/kg for Zn), *ajuru* (*Chrysobalanus ícaro*) (<0.6 mg/kg for Cu, <2.5 mg/kg for Fe, and <0.2 mg/kg for Zn), and *umari* (*Andira spinulosa*) (<0.6 mg/kg for Cu, 20.9 mg/kg for Fe, and <0.2 mg/kg for Zn) [31].

The minerals found in the pulps and flours of albino peach palm are essential for human body metabolism. Generally, the macro minerals are related to nerve cell functions, and the micro minerals are associated with the formation of erythrocyte cells [53]. Mg, the most abundant mineral found in the samples, can aid in glucose transport, control peripheral insulin resistance in type 2 diabetes mellitus, and keep tyrosine kinase activity functioning [54]. The Ca is deposited in the body as hydroxyapatite crystals in the matrix composed of collagen and directly assists in the mineralization of the skeleton [55,56]. Therefore, dietary Ca intake may directly aid in bone metabolism and development. The ingestion of RP, CP, RPF, and CPF can also supplement the daily requirement of P, which was shown to play a role in preventing sarcopenia [57] and was also related to the structural composition of teeth and bones [53]. These important elements present in 100 g of the CP supply almost 91% of the Mg, 33% of the Ca, and 23% of the P recommended for adults [58].

**Table 3:** Average contents of Al, As, Ba, Ca, Cr, Cu, Fe, Mg, Mn, P, Se, and Zn in raw and cooked albino peach palm pulp and flour.

Elements (mg/kg dm)	Pulp		Flour		Reference Values *^a^* (mg/day)
	Raw	Cooked	Raw	Cooked	
Major minerals					
Ca	1068.32 ± 137.44 ^b^	3278.34 ± 60.05 ^a^	891.70 ± 82.89 ^b^	1046.88 ± 116.06 ^b^	1000
Mg	1443.48 ± 132.16 ^b^	3632.41 ± 283.22 ^a^	1921.23 ± 192.58 ^b^	1669.45 ± 172.22 ^b^	400
P	1811.35 ± 198.27 ^a^	2237.89 ± 266.32 ^a^	1993.78 ± 19.35 ^a^	1647.83 ± 238.29 ^a^	1000
Minor minerals					
Al	23.59 ± 1.23 ^c^	24.50 ± 2.12 ^c^	89.31 ± 1.07 ^b^	127.91 ± 13.59 ^a^	
As	4.46 ± 0.20 ^a^	6.64 ± 0.67 ^a^	<LOD	<LOD	
Ba	1.11 ± 0.08 ^b^	2.39 ± 0.75 ^ab^	3.2 ± 0.14 ^a^	2.16 ± 0.16 ^ab^	
Cd	<LOD	0.09 ± <0.01	<LOD	<LOD	
Cr	20.15 ± 0.53 ^b^	21.36 ± 0.16 ^b^	1.62 ± 0.12 ^c^	32.85 ± 0.47 ^a^	
Cu	18.96 ± 0.38 ^a^	5.56 ± 1.38 ^b^	13.89 ± 0.74 ^a^	16.18 ± 1.77 ^a^	2
Fe	66.28 ± 2.30 ^a^	43.08 ± 3.96 ^b^	17.80 ± 4.49 ^b^	52.53 ± 4.49 ^ab^	18
Mn	19.34 ± 0.27 ^b^	49.61 ± 3.27 ^a^	15.35 ± 0.81 ^b^	22.17 ± 1.44 ^b^	2
Se	4.08 ± 0.57 ^a^	4.90 ± 0.24 ^a^	4.79 ± 1.40 ^a^	5.26 ± 1.15 ^a^	70 ^*b*^
Zn	9.04 ± 0.48 ^b^	22.40 ± 0.30 ^a^	16.33 ± 2.30 ^ab^	10.47 ± 1.15 ^b^	15

Mean values of three replicates ± standard deviation. Different letters on the same line represent a difference with 95% significance. Al: aluminum; As: arsenic; Ba: barium; Ca: calcium; Cr: chrome; Cu: copper; Fe: iron; Mg: magnesium; Mn: manganese; P: phosphor; Se: selenium; Zn: zinc. LOD: limit of detection. *^a^* FAO [58]; *^b^* μg/day for men and 55 μg/day for women.

### 3.4. Bioactive Compounds Contents

The contents of TPC, total flavonoids, ascorbic acid, and total carotenoids in the samples are shown in Table 4. In general, the cooking and drying processes promoted the degradation of the analyzed compounds (*p* ≤ 0.05). Regarding the cooking, the reduction observed can be attributed to the leaching of these compounds by the cooking water and the cooking temperature (~100 °C). During drying, in turn, the degradation can be attributed to the process temperature as well as the exposure time of the samples.

Rojas-Garbanzo et al. [59] were the first authors to study the effect of processing on the TPC content of peach palms. They observed 70.0 mg GAE/100 g dm in CP and 63.0 mg GAE/100 g dm in CPF. These values were higher than those observed in the CP (51.39 mg GAE/100 g) and CPF (12.16 mg GAE/100 g) of the albino peach palm. The higher TPC content found in other peach palm varieties can be attributed to the lower production of these secondary metabolites in the albino variety [60].

Flavonoids, compounds included in the group of phenolic compounds, showed similar results in the TPC during cooking, but the degradation of the TPC was 18.6%, while for total flavonoids, it was 47.3%. No significant difference (*p* > 0.05) was observed for the content of total flavonoids between the RPF and CPF, but the drying process degraded 87.7% of the flavonoids from the RP, while the CP just lost 78.8% of these compounds. These results showed that drying the peach palm pulp at 55 °C caused a greater degradation of the flavonoids in the RP than in the CP, indicating that cooking increased the thermostability of this class of compounds.

The drastic reduction in the flavonoids observed between the CP and CPF, and between the RP and RPF was different from that found for the RP and RPF concerning the TPC. This behavior can be attributed to the specificity of the Folin–Ciocalteau method for phenolics with reducing properties. Furthermore, phenolic compounds have distinct behaviors (they can be stable or associated with oxidative and enzymatic processes) when exposed to different processes such as storage, cooking, and heating [61].

Ascorbic acid (AA) showed the same behavior observed for the flavonoids during cooking. The CP showed a 19.9% reduction in the AA content compared to the RP. The AA contents in the RPF and CPF were statistically the same (*p* > 0.05). AA was shown to be susceptible to reduction at high temperatures and, as it is a water-soluble compound, it may have been leached from the pulp during cooking. However, the AA contents in the flour were approximately ten times lower than those observed in the respective pulps, which allows us to state that drying at 55 °C caused a highly significant reduction in the AA content of the raw and cooked pulps (*p* ≤ 0.05). In this way, it could be seen that the temperature and duration of drying had a much greater impact on the degradation of AA than cooking.

The AA content found by Contreras-Calderón et al. [62] in the pulp of raw peach palm (33.7 mg/100 g wb) was close to the value observed in the RP (34.38 mg/100 g dm). Prado et al. [63] found a higher AA content in raw orange peach palm flour (20.33 mg/100 g dm).

The RP and CP did not show carotenoid contents statistically different (*p* > 0.05), although there was a 6% reduction in the total carotenoid content after cooking. Regarding the flour, it was observed that the CPF had a total carotenoid content 26% higher than that observed in the RPF. This result indicated a greater fixation of the carotenoids in the CP during the drying at 55 °C. On the other hand, the results found after cooking the albino peach palm were contrary to those of some authors [64,65,66], who found higher levels of carotenoids after cooking some food matrices. These differences may be due to different botanical characteristics.

The availability of carotenoids is directly linked to the color of the fruit, the location and planting conditions, the treatment applied, the content and type of fibers, and the interaction between different carotenoids [42,59]. Because it is an albino variety, the peach palm studied was classified as a low source of carotenoids (4.92 µg β-carotene/100 g dm in RP), according to the classification proposed by Britton and Khachik [67]. This total carotenoid content was lower than that found in medlar (15.82 µg/100 g dm) [68] with whitish pulp.

### 3.5. Thermogravimetric Analysis

**Figure 1:**
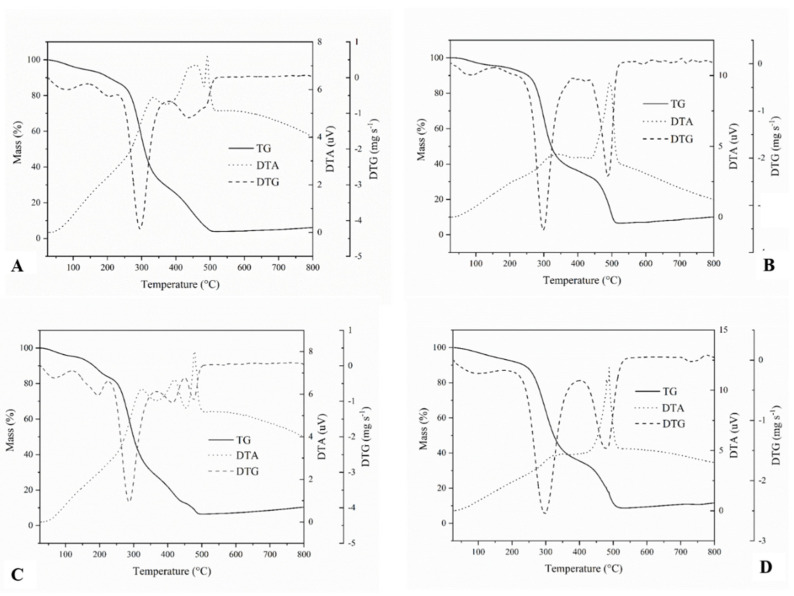
Behavior of the thermogravimetric (TG), differential thermal (DTA), and derivative thermogravimetric (DTG) curves of albino peach palm. (**A**) raw pulp; (**B**) cooked pulp; (**C**) raw pulp flour; and (**D**) cooked pulp flour.

The thermogravimetric behaviors observed for the RP, CP, RPF, and CPF of albino peach palm are shown in Figure 1. The thermogravimetric (TG) curves show the loss of mass suffered by the pulps (Figure 1A,B) and flours (Figure 1C,D), depending on the temperature, and the derivative thermogravimetric (DTG) curves show the first derivative of the TG curve. The first event observed for all the samples in the DTG curve was represented by a discrete endothermic peak, characteristic of water loss, at approximately 100 °C.

The second DTG event was an endothermic peak of greater intensity, observed for all the pulps and flours, with the onset of mass loss at 250 °C and the maximum mass loss at approximately 300 °C. This degradation pattern can be attributed to the loss of the glycosidic O-H bonds in glucose, the unit that forms amylose and amylopectin [69,70,71]. Similar behavior was observed for peach palm native starch, which showed a degradation peak, starting at 251.7 °C and a maximum weight loss at 320 °C [70].

In the differential thermal analysis (DTA), two exothermic peaks were observed for all products (pulps and flours), the first at approximately 350 °C and the second at approximately 500 °C. These peaks are related to the release of energy in the same region of the main mass decays of the TG/DTG curves. The first peak showed a low intensity for the RP (Figure 1A) and RPF (Figure 1C) and was practically suppressed for the CP (Figure 1B) and CPF (Figure 1D). On the other hand, the second peak showed a low intensity for the RP (Figure 1A), a moderate intensity for the RPF (Figure 1C), and a higher intensity for the CP (Figure 1B) and CPF (Figure 1D).

The exothermic peaks observed for the RP were related to the release of energy resulting from successive degradations and oxidations of organic-based macronutrients (proteins, carbohydrates, lipids, and fibers) and inorganic macro- and microminerals. On the other hand, for the CP, RPF, and CPF, the increase in the intensity, mainly of the second peak, was attributed to the release of energy from the carbonization process, since these products were submitted to thermal processes (cooking and/or drying), which can promote starch gelatinization, protein denaturation, and lipid oxidation [72].

The exothermic peak at approximately 500 °C paralleled a mass decay observed in the TG curve, representing the final mass loss. Finally, the thermal behavior presented by the RP and CP indicated that the hydrothermal treatment to which the fruits were submitted (cooking with water at high temperature and pressure) and drying at 55 °C, used to obtain the flour, interfered with the differential thermogravimetry profiles of the products.

### 3.6. Absorption Spectroscopy in the Infrared Region

**Figure 2:**
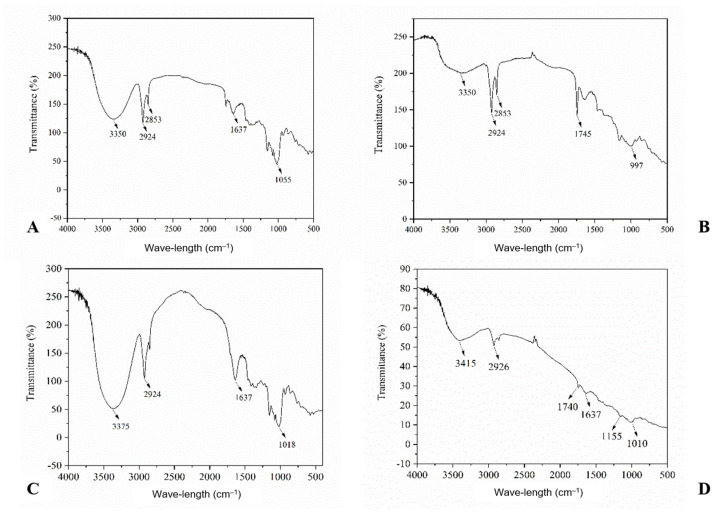
Infrared spectroscopy of albino peach palm. (**A**) raw pulp; (**B**) cooked pulp; (**C**) raw pulp flour; (**D**) cooked pulp flour.

Figure 2 shows the Fourier transform infrared spectroscopy (FTIR) results for the RP, CP, RPF, and CPF. The spectral patterns presented in the evaluated frequency range evidenced the presence of peaks between 3350 and 1055 cm^−1^ for the RP (Figure 2A); 3350 and 997 cm^−1^ for the CP (Figure 2B); 3375 and 1018 cm^−1^ for the RPF (Figure 2C); and between 3415 and 1010 cm^−1^ for the CPF (Figure 2D). In general, the peaks detected for the pulps (RP and CP) and flours (RPF and CPF) were similar to those found by Giombelli et al. [40] for the dietary fiber concentrates from peach palms obtained via different extraction procedures.

The spectra of the RP (Figure 2A) and CP (Figure 2B) exhibited a medium width and medium absorption peak at 3350 cm^−1^, while the RPF (Figure 2C) showed a broad and medium absorption band at 3375 cm^−1^. This band is characteristic of hydrogen bonds, with the presence of the O–H of hydroxyl groups, present in the D-glucose units and their glycosidic bonds, units that constitute the starch structures of plant species [72], like peach palms. On the other hand, the CPF (Figure 2D) had a peak at 3415 cm^−1^, with stretching characteristic of primary and secondary amines and amides (N–H).

The RP and RPF showed a strong absorption peak at 1637 cm^−1^, which was practically suppressed in the CP and CPF. This peak is characteristic of the presence of primary amide bonds (primary elongation C=O). For secondary amides, the characteristic band occurred at 1155 cm^−1^, which is present in proteins [73,74]. These results were consistent with the lower protein content observed in the CP compared to the RP (Table 1).

The four samples showed medium and low absorption peaks between 2926 and 2853 cm^−1^, which correspond to the stretching of alkanes (–CH_3_) and the stretching of the bonds of the C–O glycosidic groups, related to the bonds of the starch-forming units (D-glucose molecules) [74,75]. Peaks in this absorption range were detected in the flour from other peach palm varieties [14].

The observed absorptions at 1740 and 1745 cm^−1^ for the CP (Figure 2B) and CPF (Figure 2D) were attributed to the C=O stretching of carbonyl esters present in functional groups such as ketones and aldehydes. The fact that these peaks were revealed only for the CP and CPF indicates that the hydrothermal process promoted the release of proteins in the albino peach palm.

The absorption bands at 1055 cm^−1^ for the RP and 997 cm^−1^ for the CP are characteristic of alcohols, esters, ethers, and carboxylic acids, among other functional groups. The smallest band at 997 cm^−1^ can be attributed to the sequence of the aliphatic chains and aromatic rings of fatty acids in PC [5,73,74]. These peaks were also observed by Pires et al. [14] for flour from other peach palm varieties.

The interaction of the hydrogen bonds of water with the glycosidic bonds (α 1-4 and α 1-6) of the starch-forming units (D-glucose units) can cause changes in the linear (amylose) and branched (amylopectin) chains of starch. These alterations can promote structural and functional changes in starch, attributed to gelation, paste formation, and retrogradation [70,76].

### 3.7. Morphological Aspects

**Figure 3:**
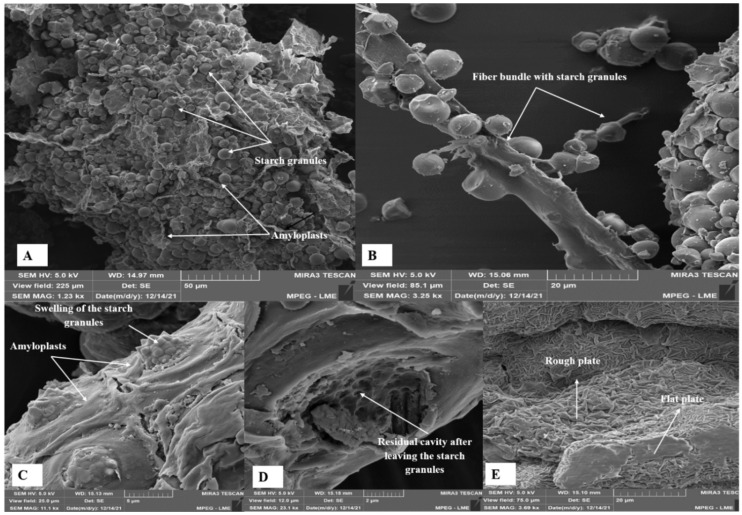
Micrographs of the raw pulp of albino peach palm: (**A**) 1.23 k× resolution and (**B**) 3.25 k× resolution; cooked pulp: (**C**) 11.1 k× resolution; (**D**) 23.1 k× resolution; (**E**) 3.69 k× resolution.

**Figure 4:**
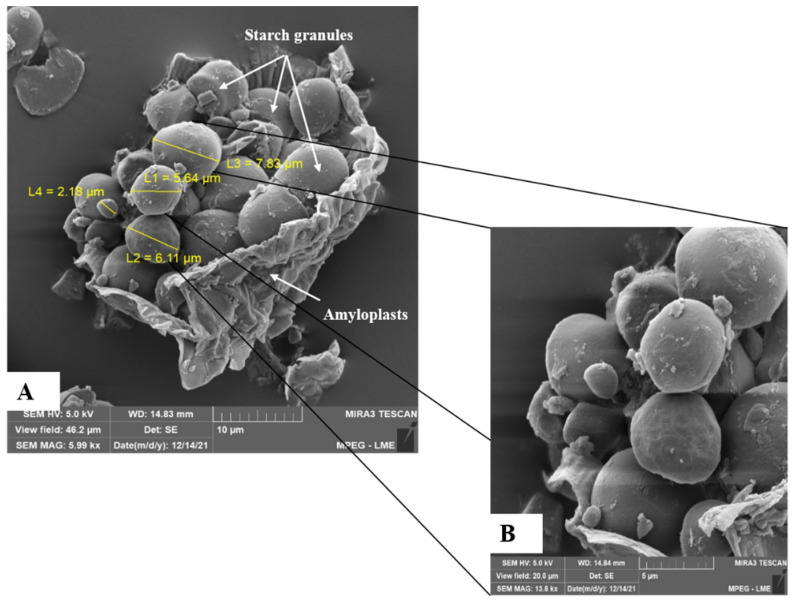
Micrographs of raw pulp flour of albino peach palm. (**A**) at 5.99 k× resolution and (**B**) 13.8 k× resolution.

**Figure 5:**
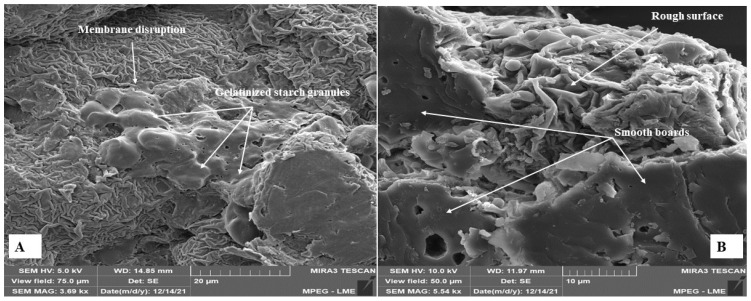
Micrographs of the cooked pulp flour of albino peach palm. (**A**) at 3.69 k× resolution and (**B**) 5.54 k× resolution.

Scanning electron microscopy (SEM) was used to evaluate whether the hydrothermal process (cooking in water under high temperature and pressure) applied to peach palm and if drying the RP and CP at 55 °C promoted morphological structure changes in the CP, RPF, and CPF. The micrographs are shown in Figure 3, Figure 4 and Figure 5. Figure 3A presents an overview of the RP, highlighting the plastids or amyloplasts, which are non-pigmented organelles specialized in synthesizing starch via the polymerization of glucose molecules, storing them in the form of starch granules (amyliferous) [77]. The presence of fibrous structures in bundles with attached starch granules (Figure 3B) may indicate functions other than energy, as starches that participate in this type of coupling are considered resistant and perform functions similar to fibers [78]. Thus, part of the starches attached to the fiber bundles may not be digested by the action of enzymes in the small intestine, which can promote beneficial effects on the human body, such as accelerating the digestion process, increasing satiety, and the consequent loss of weight [79,80].

The micrographs showed that the CP’s morphological structure had undergone more modifications compared to the RP. During the hydrothermal process, heat in the presence of water promoted the rupture of amyloplasts, with the consequent release of some starch granules (Figure 3C). This destruction of the parenchymal architecture promoted the formation of residual spongy structures in the membranes (Figure 3D). Figure 3E shows an intense disruption of the parenchyma architecture due to ruptured amyloplasts, which is attributed to starch gelatinization during the hydrothermal process and to a probable retrogradation effect after cooking [72]. There was a loss of the compartmentalization of the structures, resulting in the stacking of plates with rough membranes and some smooth plates.

The micrographs of the RPF (Figure 4) showed the presence of an amyloplast containing multiple heterogeneous starch grains, with diameters between 2.18 µm and 7.83 µm and predominantly oval and spherical shapes, with a smooth surface and no cracks. The granule shapes that remained after drying at 55 °C compared to the RP (Figure 3A,B) were similar to the profiles observed by Ferrari Felisberto et al. [69] for the starch granules from another peach palm variety. Lindeboom et al. [72] proposed a classification for starch granules, according to their size, as very small (<5 µm), small (5–10 µm), medium (10–25 µm), and large (>25 µm). Based on this classification, the peach palm starch granules were classified as small (5–10 µm). Neto et al. [70] reported the diameters of the same order of magnitude (1.6–8.5 μm) for the starch granules from a peach palm species, while Valencia et al. [76] observed larger starch granules (5.2–12.5 µm) in the peach palm flour of the red variety. Knowing the structures of the starch and the size of the granules is relevant for defining the applications for starch, since these properties can interfere with digestion after cooking (contact surface), water retention, and starch efficiency as an encapsulating agent [70].

In general, the morphological aspects of the CPF (Figure 5) were similar to those observed for the CP (Figure 3C–E), confirming that the main morphological changes occurred during hydrothermal cooking. Torres-Vargas et al. [43] observed that green peach palm flour, raw and cooked under pressure (121 °C) for 1 h, showed the same botanical architecture for the starch granules, but there was also a disruption of the amyloplast membranes.

### 3.8. Particle size Analysis of Flour

**Figure 6:**
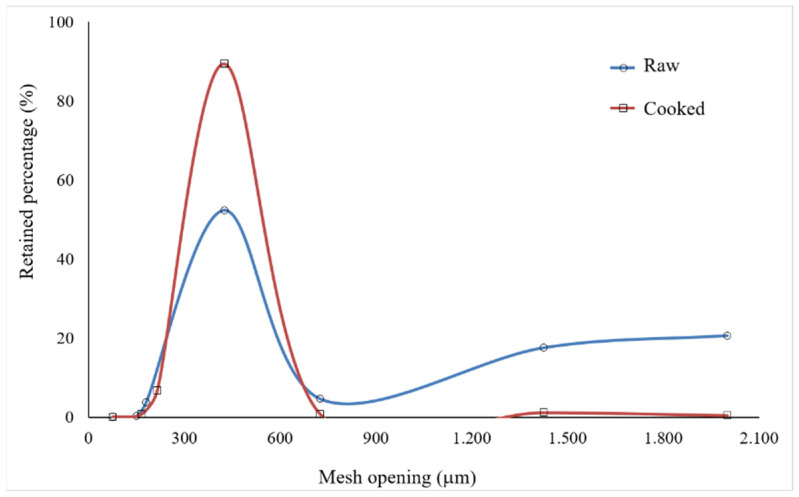
Grain size distribution for raw pulp flour and cooked pulp flour of albino peach palm.

The results of the particle size distribution for both the RPF and CPF (Figure 6) showed that the flour had slightly different granulometric profiles, but the highest retention occurred in the mesh with an opening of 425 μm for both flour. On the other hand, 89.4% of the CPF was retained on this sieve, while the retention was only 52.4% for the RPF. Regarding the remaining fraction, the largest amount of RPF (43%) had an average particle size greater than 425 μm, while the largest amount of CPF (~8%) had an average particle size smaller than 425 μm.

As the conditions used in drying the RP and CP and in grinding the dry products were the same, the granulometric profiles of the flour allow us to state that the hydrothermal process used in cooking the peach palm caused the weakening of the molecular structures of the albino peach palm pulp, making the CPF less resistant to milling than the RPF. The observed behavior can be attributed to the disruption of the amyloplast membranes during the hydrothermal process, evidenced in the micrographs of the CP (Figure 3) and CPF (Figure 5). The fact that the granulometric distributions of both flour presented a single peak, with the same amplitude and great intensity indicated that the flour presented uniform granulometry. However, the peak of greater intensity observed for the CPF allows us to state that this flour had a greater granulometric uniformity, which directly interfered with its sensory quality (texture, flavor, and appearance) [81].

### 3.9. Technological Functional Properties

The values of the technological functional properties analyzed in the RPF and CPF are shown in Table 5. The CPF had a lower water solubility index (WSI) and oil absorption capacity (OAC), and a higher water absorption index (WAI) than the RPF (*p* ≤ 0.05). These results indicated that the hydrothermal process (cooking) caused changes in the main technological functional properties of the albino peach palm flour. Zhou et al. [82] reported that the WSI of starchy matrices was related to the presence of starch and soluble proteins after cooking in the presence of water. According to Hatamian et al. [83], cooking promoted damage to starch granules and, consequently, an increase in the WSI. This behavior was not observed for the albino peach palm under the conditions studied and can be attributed to the leaching of water-soluble constituents from the pulp, such as sugars (Table 1), during the hydrothermal process.

Regarding the WAI, according to Silva [81], flour with a smaller granulometry adsorbs more water than flour with larger particle sizes, which is in agreement with the results observed in the present study, in which the CPF that presented a greater number of smaller particles (Figure 6), was the one with the highest WAI value (*p* ≤ 0.05). Additionally, the highest WAI value for the CPF indicated that during the hydrothermal process, starch molecules might have been converted into lower-molecular-weight molecules (e.g., dextrins), which have more affinity for water molecules [83,84].

The results showed that during the hydrothermal process, there was a greater increase in the hydrophilic constituents than in the hydrophobic constituents in the pulp, which justifies a lower OAC and a higher WAI in the CPF. The OAC value is directly related to the protein content in the food; therefore, the lower the protein content, the lower the food’s ability to absorb oil [85]. The OAC is an index to be considered when using flour in formulations to improve their sensory characteristics [86]. Proteins present on the surface of the product increase the hydrophobic interaction with flavor compounds [87]. However, the albino peach palm presented behavior different to what was expected (Table 1).

Finally, the RPF showed foaming capacity (FC), which indicates that the applied cooking eliminated the foaming compounds from the pulp. Likewise, proteins also influence the foaming capacity of the flour due to their amphiphilic properties and because they are surfactants [88]. The ability of flour to form foam directs its use in manufacturing products to improve the texture, consistency, and appearance, mainly of desserts and cocktails [89].

## 4. Conclusions

This study comprises the first report on the chemical composition, bioactive compounds, and physicochemical, functional–technological, color, thermal, and morphological properties of the raw pulp (RP), cooked pulp (CP), raw pulp flour (RPF), and cooked pulp flour (CPF) of albino peach palm. The results showed that the hydrothermal treatment (cooking) decreased the contents of lipids, total dietary fiber, and sugars of the albino peach palm pulp, while drying at 55 °C caused the concentration of these nutrients in the flour (except for carbohydrates and sugar). Cooking and drying, in turn, caused the browning of the CP and CPF. The RP turned out to be an excellent source of calcium, magnesium, and phosphorus, contributing to the requirements for human health, even though this latter mineral was decreased by cooking. The contents of phenolic compounds, flavonoids, ascorbic acid, and carotenoids were negatively impacted by cooking. The thermal analysis showed that the cooking and drying processes modified the thermogravimetric profile of the products, while the FTIR showed that there were little changes in the functional groups of the chemical structures of the pulp after cooking, which was more representative in the dry pulp (flour). The morphological aspects proved the existence of fibrous structures, including those linked to starch granules in the albino peach palm pulp, and evidence of the starch gelatinization caused by cooking. The CPF had smaller particle sizes than the RPF, showing that cooking caused the softening of the more resistant structures of the albino peach palm pulp. On the other hand, the RPF had a higher water solubility index, oil absorption capacity, and foam stability, and lower water absorption index than the CPF. The results of this research should encourage studies on the other components of the albino peach palm, as well as the technological and functional application of albino peach palm flour to enrich foods, especially in dietary fibers, calcium, magnesium, and phosphorus. In addition, it is also important to better explore the bioactive potential of extracts from this peach palm variety in biological assays.

## Figures and Tables

**Table 1:** Proximate composition and physicochemical properties of raw and cooked albino peach palm pulp and the respective flour.

Components	Pulp		Flour	
	Raw	Cooked	Raw	Cooked
Moisture (g/100 g wet basis—wb)	61.00 ± < 0.01 ^b^	70.50 ± 0.50 ^a^	9.67 ± 0.29 ^c^	5.83 ± 0.29 ^d^
Ash (g/100 g dry matter—dm)	9.68 ± 1.30 ^b^	13.93 ± 0.65 ^a^	2.21 ± < 0.01 ^c^	2.12 ± < 0.01 ^c^
Proteins (g/100 g dm)	7.90 ± < 0.01 ^c^	7.59 ± 0.23 ^c^	16.96 ± < 0.01 ^b^	19.99 ± 0.45 ^a^
Lipids (g/100 g dm)	10.21 ± 1.12 ^a^	8.63 ± 0.84 ^a^	10.81 ± 0.31 ^a^	7.86 ± 0.52 ^a^
Total carbohydrates (g/100 g dm)	72.08 ± 2.94 ^a^	70.58 ± 0.14 ^a^	69.92 ± 0.69 ^a^	69.59 ± 0.53 ^a^
Total dietary fiber (g/100 g dm)	13.64 ± 2.27 ^ab^	12.81 ± 2.18 ^ab^	16.89 ± 0.84 ^a^	8.19 ± 0.19 ^b^
Insoluble dietary fiber (g/100 g dm)	9.30 ± 0.72 ^b^	7.32 ± 0.01 ^b^	14.72 ± 0.70 ^a^	6.03 ± 1.38 ^b^
Soluble dietary fiber (g/100 g dm)	5.41 ± 1.85 ^ab^	6.74 ± 0.18 ^a^	2.66 ± 0.11 ^b^	2.16 ± 0.19 ^b^
Total sugars (g/100 g dm)	59.18 ± 1.11 ^a^	49.10 ± 0.57 ^b^	24.74 ± 0.45 ^c^	12.46 ± 0.12 ^d^
Reducing sugars (g/100 g dm)	12.58 ± 0.21 ^a^	3.86 ± < 0.01 ^c^	12.92 ± < 0.01 ^a^	6.98 ± 0.29 ^b^
Non-reducing sugars (g/100 g dm)	46.60 ± 1.02 ^a^	45.24 ± 0.61 ^b^	11.82 ± 0.45 ^c^	5.48 ± 0.26 ^d^
Total energetic value (kcal/100 g wb)	160.58 ± 0.50 ^b^	115.19 ± 1.20 ^c^	401.85 ± 0.10 ^a^	400.00 ± 1.70 ^a^
a_w_	0.980 ± < 0.01 ^a^	0.981 ± < 0.01 ^a^	0.206 ± < 0.01 ^c^	0.246 ± < 0.01 ^b^
pH	6.44 ± 0.08 ^a^	6.18 ± 0.03 ^a^	6.15 ± 0.05 ^b^	6.25 ± 0.13 ^a^
Total titratable acidity (g citric acid/100 g wb)	8.96 ± < 0.01 ^c^	4.48 ± < 0.01 ^d^	21.32 ± 0.30 ^b^	23.45 ± 0.60 ^a^

Mean values of three replicates ± standard deviation. Different letters on the same line represent differences with 95% significance for Tukey’s test (*p* ≤ 0.05). a_w_: water activity.

**Table 2:** Color parameters for raw and cooked albino peach palm pulp and flour.

Parameter	Pulp		Flour	
	Raw	Cooked	Raw	Cooked
L*	76.52 ± 4.78 ^a^	70.37 ± 1.26 ^b^	80.23 ± 2.2 ^a^	72.03 ± 1.61 ^b^
a*	−0.43 ± 0.21 ^a^	−1.59 ± 0.37 ^b^	−1.39 ± 0.04 ^b^	−0.47 ± 0.12 ^a^
b*	+18.56 ± 0.86 ^b^	+25.88 ± 0.67 ^a^	+16.0 ± 0.72 ^b^	+24.93 ± 1.66 ^a^
C*	18.57 ± 0.86 ^b^	25.93 ± 0.69 ^a^	15.17 ± 0.1 ^c^	24.93 ± 1.64 ^a^
*h°*	91.35 ± 0.75 ^b^	93.50 ± 0.73 ^a^	94.92 ± 0.02 ^a^	90.94 ± 0.27 ^b^
WI	70.00 ± 3.46 ^a^	60.60 ± 0.43 ^b^	74.58 ± 0.61 ^a^	62.48 ± 0.23 ^b^

Mean values of three replicate ± standard deviation. Different letters on the same line represent a difference, with 95% significance (*p* ≤ 0.05). WI: whiteness index.

**Table 4:** Composition of bioactive compounds in the raw and cooked albino peach palm pulp and flour.

Components	Pulp		Flour	
	Raw	Cooked	Raw	Cooked
TPC (mg GAE/100 g dry matter—dm)	63.10 ± 3.10 ^a^	51.39 ± 5.47 ^b^	48.47 ± 0.98 ^b^	12.16 ± 2.27 ^c^
Total flavonoids (mg quercetin/100 g dm)	19.31 ± 0.13 ^a^	10.17 ± 0.42 ^b^	2.37 ± 0.18 ^c^	2.16 ± < 0.01 ^c^
Ascorbic acid (mg/100 g dm)	88.15 ± < 0.1 ^a^	70.63 ± 6.13 ^b^	7.90 ± < 0.1 ^c^	6.06 ± < 0.1 ^c^
Total carotenoids (μg β-carotene/100 g dm)	4.92 ± 0.20 ^ab^	4.62 ± 0.65 ^ab^	4.39 ± 0.02 ^b^	5.53 ± 0.21 ^a^

Mean values of three replicates ± standard deviation. Different letters on the same line represent a difference, with 95% significance (*p* ≤ 0.05). TPC: total phenolic compound; GAE: gallic acid equivalent.

**Table 5:** Functional technological properties of raw pulp and cooked pulp flour of albino peach palm.

Property	Flour	
	Raw Pulp	Cooked Pulp
WSI (%)	26.57 ± < 0.01 ^a^	18.05 ± 1.84 ^b^
WAI (g/g dry matter)	1.46 ± 0.04 ^b^	2.07 ± 0.08 ^a^
OAC (g/g dry matter)	1.04 ± 0.04 ^a^	0.89 ± 0.03 ^b^
FC (%)	11.11 ± 2.24	nr
FS	nr	nr

Mean values of three replicates ± standard deviation. Different letters on the same line represent a difference with 95% significance. WSI: water solubility index; WAI: water absorption index; OAC: oil absorption capacity; FC: foaming capacity; FS: foam stability; nr: no response for method.

## Data Availability

Data are contained within the article.

## Supplementary Materials

The following supporting information can be downloaded at: https://www.mdpi.com/article/10.3390/foods12234344/s1.
