# Supplementary material for: Nutritional and Technological Properties of Albino Peach Palm (Bactris gasipaes) from the Amazon: Influence of Cooking and Drying
Source: Foods. 2023 Dec 1;12(23):4344. doi: 10.3390/foods12234344 (PMC10706845; doi:10.3390/foods12234344)
Supplement: Supplementary file 1 [file foods-12-04344-s001.zip › foods-2723476-supplementary.pdf]

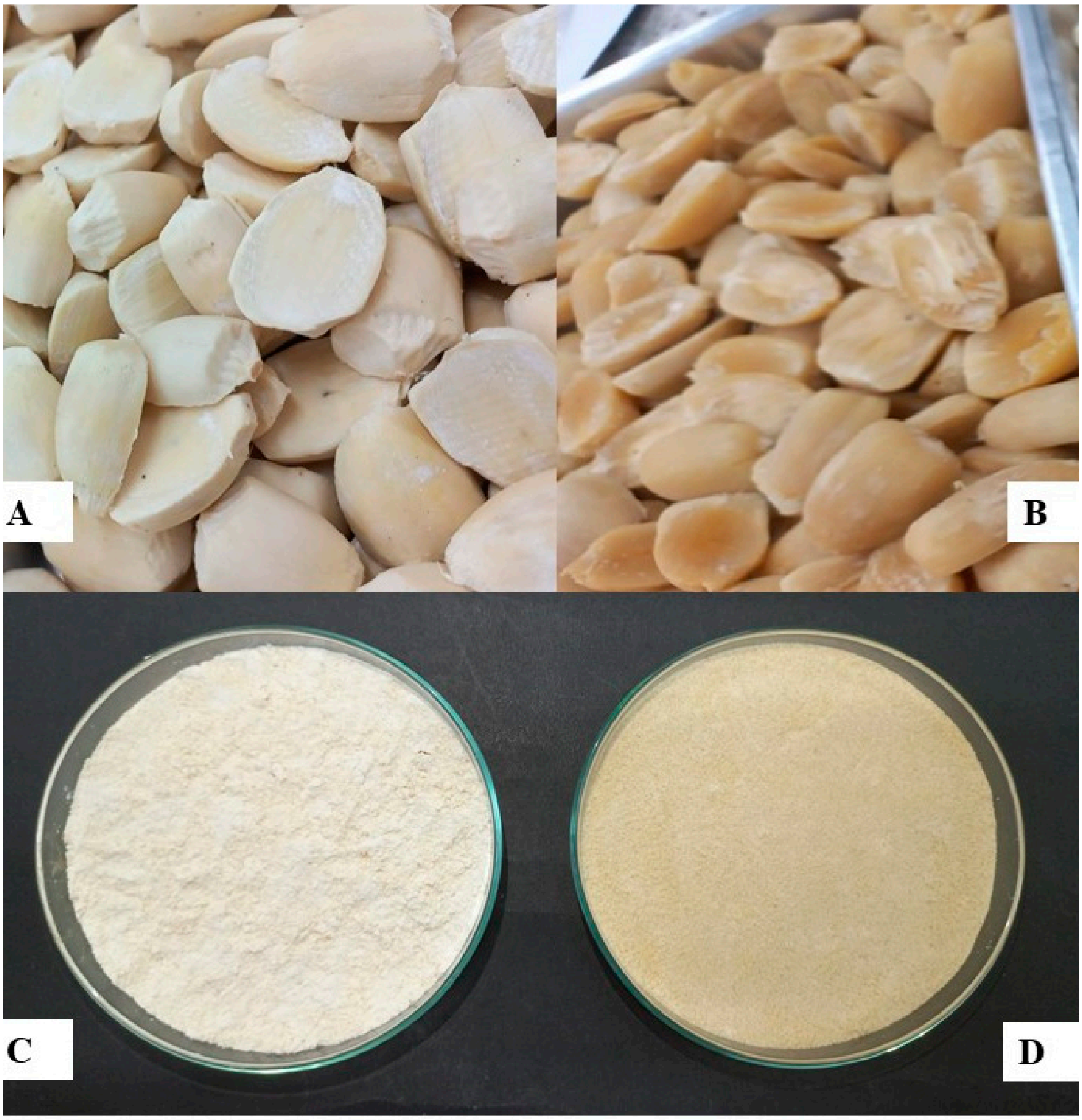


**Figure S1.** Visual appearance of the albino peach palm: (A) raw pulp; (B) cooked pulp; (C) raw pulp flour; (D) cooked pulp flour.
